# Causal Relationship Between Brain Functional Networks and Sleep Disorders: A Mendelian Randomization Study

**DOI:** 10.1002/brb3.70870

**Published:** 2025-10-07

**Authors:** Wenting Lin, Xiaoqin Chen, Yuxing Wei, Lu Yang, Hui Li, Tianmin Zhu

**Affiliations:** ^1^ School of Rehabilitation and Health Preservation Chengdu University of Traditional Chinese Medicine Chengdu Sichuan China; ^2^ Chengdu Pidu District Hospital of Traditional Chinese Medicine Chengdu Sichuan China; ^3^ School of Acupuncture and Tuina Chengdu University of Traditional Chinese Medicine Chengdu Sichuan China; ^4^ School of Preclinical Medicine Chengdu University Chengdu Sichuan China

**Keywords:** addictive behaviors, causal association, Mendelian randomization, resting‐state functional magnetic resonance imaging, sleep disorders

## Abstract

**Background:**

Dysfunction of brain resting‐state functional networks has been widely reported in sleep disorders, but their causal relationships remain controversial.

**Objective:**

To investigate causal relationships between 191 resting‐state functional magnetic resonance imaging (rsfMRI) phenotypes and eight sleep disorders using bidirectional two‐sample Mendelian randomization (MR), with multivariable MR (MVMR) adjusting for addictive behaviors.

**Methods:**

Genome‐wide association study (GWAS) data from UK Biobank and FinnGen were analyzed. Instrumental variables (IVs) were selected adhering to MR assumptions. Causal estimates were primarily derived via inverse‐variance weighted (IVW), with sensitivity analyses using MR‐Egger, weighted median, weighted mode, simple mode, and MR Pleiotropy Residual Sum and Outlier (MR‐PRESSO).

**Results:**

We found increased functional connectivity within the salience and default mode networks in insular, cingulate, and frontal regions​​ (OR = 0.94, 95% CI: 0.92–0.97, pFDR = 0.042) was causally associated with a 6% reduced risk of daytime napping. For obstructive sleep apnea (OSA), ​​elevated functional connectivity within the default mode and central executive networks localized to precuneus/occipital regions​​ (OR = 1.16, 95% CI: 1.08–1.25, pFDR = 0.042) and ​​elevated functional connectivity within the default mode and central executive networks localized to parietal/temporal regions​​ (OR = 1.18, 95% CI: 1.09–1.27, pFDR = 0.016) having a causal effect, associated with a 16% and 18% elevated risk of OSA respectively. MVMR analysis revealed that coffee intake did not confound associations between rsfMRI phenotypes and sleep disorders, while smoking, alcohol consumption, tea intake, and cannabis use may act as confounding factors affecting these links.

**Conclusion:**

This study provides robust evidence for causal associations between specific rsfMRI phenotype signatures and sleep disorders. It highlights brain functional networks as potential targets for non‐invasive interventions, particularly within the context of addictive behaviors.

## Graphical abstract

This Mendelian randomization study establishes causal links between specific brain functional networks and sleep disorders. Enhanced connectivity in salience/default mode networks reduces daytime napping risk, while elevated activity in default/executive networks increases obstructive sleep apnea risk. Addictive behaviors (smoking, alcohol, cannabis, tea) act as significant confounders in these brain–sleep relationships.

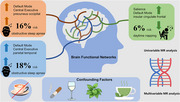

## 1 Introduction

Sleep critically influences cognitive and emotional well‐being through its duration, quality, and circadian regulation (Monk and Welsh 2003). The dysregulation of sleep in neuropsychiatric disorders, such as Major Depressive Disorder (Franzen and Buysse 2008) and Alzheimer's disease (Z. Wang et al. 2022), further underscores its importance. Notably, these disorders frequently co‐occur with measurable alterations in brain structure or function. For Major Depressive Disorder, accumulating evidence has documented changes in brain regions such as the prefrontal cortex and hippocampus (Desseilles et al. 2008), while for Alzheimer's disease, it is characterized by well‐established cortical atrophy and gray matter volume reduction (Lv et al. 2023). These observations collectively suggest potential links between sleep disorder and underlying neural mechanisms.

Functional brain networks represent high‐dimensional architectures that underpin numerous advanced cognitive functions of the human brain. Specifically, these networks serve as the neurobiological substrate for the brain's complex operations, with inter‐network connections and communication forming the basis of cognition, behavior, and emotion (Park and Friston 2013). The human brain maintains spontaneous neural activity even in a resting state, and resting‐state functional magnetic resonance imaging (rsfMRI)—a valuable non‐invasive technique—enables the visualization of functionally active brain regions during this state. Functional magnetic resonance imaging (fMRI) studies have identified a variety of intrinsic resting‐state networks (RSNs) in the brain, including the salience network (SN), default mode network (DMN), central executive network (CEN), and attention network, among others (Finn et al. 2015; Yeo et al. 2011). Mounting evidence indicates that alterations in functional connectivity within these RSNs are closely associated with sleep disturbances (Taebi et al. 2024; Taki et al. 2012). For instance, rsfMRI investigations have revealed significant changes in the DMN and other core RSNs in individuals with insomnia and obstructive sleep apnea (OSA) (de Faria et al. 2020; Hramov et al. 2020; Y. Liu et al. 2024), highlighting their potential roles in the pathophysiology of these sleep disorders. Despite these insights, critical ambiguities persist in clarifying their causal interplay. The direction of causality between sleep disorders and rsfMRI phenotypes remains undefined, whether sleep disorders drive brain functional changes or vice versa. Further, whether addictive behaviors act as potential confounding variables that independently influence both brain functional networks and sleep disorders remains unclear. Crucially, observational studies are limited by confounding factors and reverse causality, making it difficult to establish definitive causal links. Thus, the causal pathways linking sleep disorders and brain functional networks, and the role of addictive behaviors as independent risk factors or mediators, remain unresolved.

Genome‐wide association study (GWAS) has proven highly effective in identifying single nucleotide polymorphisms (SNPs) causally associated with common complex diseases (Mallard et al. 2023), providing valuable insights into their genetic architecture. Mendelian randomization (MR) leverages genetic variants, identified by GWAS, as instrumental variables (IVs) to investigate causal relationships between risk factors and disease outcomes (Weith and Beyer 2023). By leveraging the random assignment of genetic variants before disease onset, MR analysis can exclude confounding factors and identify causal determinants of specific outcomes (X. Liu et al. 2022).

Given the unresolved causal pathways between sleep disorders and brain functional networks, and the unclear role of addictive behaviors in this interplay, we hypothesized that specific rsfMRI phenotypes exert direct causal effects on sleep disorders, while addictive behaviors, including cigarette smoking, alcohol consumption, tea intake, coffee intake, and cannabis use, may act as potential confounders. We aimed to first identify causal links between 191 rsfMRI phenotypes and 8 sleep disorders (including insomnia, daytime napping, sleep duration, long sleep, short sleep, chronotype, snoring, and OSA) using Univariable Mendelian randomization (UVMR), and then use Multivariable MR (MVMR) to adjust for these addictive behaviors as potential confounders, thereby isolating the independent direct effects of rsfMRI phenotypes by accounting for their confounding influence. By clarifying these causal pathways and the role of addictive behaviors, we seek to provide insights into underlying mechanisms and emphasize the importance of considering such behaviors in future research and clinical interventions.

## 2 Methods

### 2.1 Study Design Overview

**FIGURE 1:**
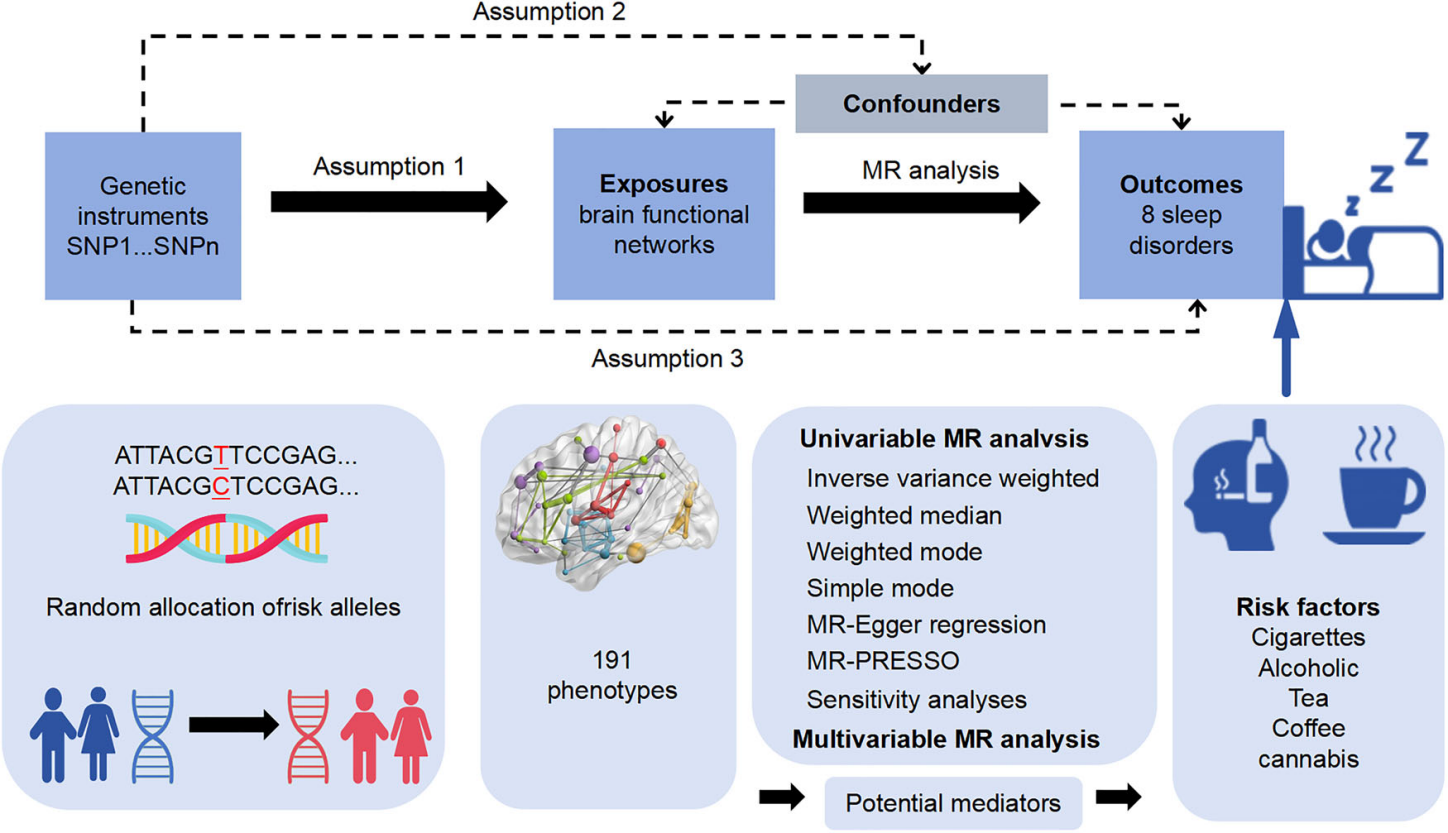
Study design overview. MR, Mendelian randomization; MR‐PRESSO, Mendelian Randomization Pleiotropy Residual Sum and Outlier; SNP, single nucleotide polymorphism.

This MR study, outlined in Figure 1, utilized GWAS summary statistics directly sourced from institutional repositories, including UK Biobank (UKB), FinnGen, and GSCAN consortium, to explore the causal links between brain functional networks and sleep disorders. Initially, UVMR was employed to evaluate the direct causal impacts of brain rsfMRI phenotypes on sleep disorders. Subsequently, within the risk factor analysis, potential mediators related to brain functional networks were scrutinized and then validated through MVMR. This method of using IVs, which mimics the random allocation of SNPs in offspring, is comparable to the setup of a randomized controlled trial (RCT). Only genetic variants that satisfied these criteria were selected as IVs: a robust connection with the exposure, no association with confounding variables, and no direct influence on the outcome (Hartwig et al. 2016). All the analyses were carried out in line with the STROBE‐MR guidelines (Skrivankova et al. 2021).

### 2.2 Data Sources

#### 2.2.1 GWASs of Brain rsfMRI

This study utilized rsfMRI datasets sourced from previous research conducted by Zhao et al. (2022), which explored links across 1,777 intrinsic brain activity phenotypes and 9,026,427 common genetic variants in a UKB cohort of 34,691 individuals. Owing to documented evidence that genetic impacts on functional neural networks tend to be less pronounced than structural ones, as indicated by Elliott et al. (2018) (Grasby et al. 2020; Zhao et al. 2019), the original traits were filtered to include only phenotypes demonstrating significant genetic associations, defined by a *p* value threshold of less than 5 × 10^−8^. Following this step, 191 traits were selected for GWAS analysis; this set comprised 75 amplitude‐related measures (representing nodes of regional spontaneous activity), 111 pairwise connectivity scores (edges), along with five metrics for global functional connectivity. These phenotypic variables extend across multiple brain systems, namely the SN, DMN, CEN, somatomotor, attention, limbic, and visual networks. For full dataset specifications, refer to the primary publication and Table S1.

#### 2.2.2 Sleep Disorders

Genetic association data for the following sleep disorders were procured from GWASs conducted in the UKB: chronotype (*n* = 449,734 individuals) (Jones et al. 2019), daytime napping (*n* = 452,633 individuals) (Dashti et al. 2021), sleep duration (*n* = 446,118 individuals) (Dashti et al. 2019), long sleep duration (*n* = 33,926 individuals, comprising 34,184 cases and 305,742 controls) (Dashti et al. 2019), short sleep duration (*n* = 411,934 individuals, including 106,192 cases and 305,742 controls) (Dashti et al. 2019), insomnia (*n* = 208,958 individuals, with 66,976 cases and 141,982 controls) (Watanabe et al. 2022), and snoring (*n* = 218,346 individuals, consisting of 61,792 cases and 156,554 controls) (Campos et al. 2020). Genetic association estimates for OSA were obtained from the FinnGen consortium's (Kurki et al. 2023) ninth data release of GWAS summary statistics, dated June 24, 2024. Detailed specifications for these datasets are provided in the original publications and Table 1.

**TABLE 1:** Summary of genome‐wide association studies used.

Trait	PubMed ID	Consortium	Sample sizes	Ancestry
**Outcome**				
Insomnia	34594039 Sakaue et al. (2021)	UK Biobank	669,760 cases 141,982 controls	European
Daytime nap	33568662 Dashti et al. (2021)	UK Biobank	425,633	European
Sleep duration	30846698	UK Biobank	446,118	European
Sleep duration (short sleep)	30846698 Dashti et al. (2019)	UK Biobank	106,192 cases 305,742 controls	European
Sleep duration (long sleep)	30846698	UK Biobank	34,184 cases 305,742 controls	European
Chronotype	30696823 Jones et al. (2019)	UK Biobank	449,734	European
Snoring	32060260 Campos et al. (2020)	UK Biobank	61,792 cases 156,554 controls	European
Sleep apnea		FinnGen	38,998 cases 336,695 controls	European
**Covariate**				
Cigarettes per day	30643251 Liu et al. (2019)	GSCAN	337,334	European
Alcoholic drinks	30643251	GSCAN	941,280	European
Tea intake		UK Biobank	447,485	European
Coffee intake		UK Biobank	428,860	European
Ever taken cannabis	30150663 Pasman et al. (2018)	UK Biobank	184,765	European

Abbreviation: GSCAN, GWAS and Sequencing Consortium of Alcohol and Nicotine use;GWAS, genome‐wide association study.

#### 2.2.3 Potential Mediators

IVs for cigarette and alcohol consumption were sourced from GWAS conducted by the GSCAN consortium (Tan et al. 2024), which identified 23 SNPs associated with daily cigarette use (*n* = 337,334) and 35 SNPs linked to weekly alcohol intake (*n* = 941,280) in European populations (M. Liu et al. 2019). Genetic instruments for addictive behavioral variables, including tea and coffee intake, were derived from the UKB, encompassing 447,485 and 428,860 European participants, respectively. Detailed phenotype definitions for these addictive behaviors are available in the UKB's official database (https://biobank.ndph.ox.ac.uk/showcase/search.cgi). In addition, genetic variants associated with lifetime cannabis use were obtained from a UKB GWAS involving 184,765 European individuals (Pasman et al. 2019). All genetic instruments underwent harmonization to exclude variants absent in the outcome GWAS datasets, with no proxy replacements employed.

#### 2.2.4 Selection of the Genetic Instrumental Variables

To satisfy the three fundamental assumptions underpinning MR, IVs underwent rigorous pre‐analytical quality control. The initial selection criterion required SNPs to exhibit genome‐wide significant associations (*p* < 5 × 10^−8^) with the target exposure. Linkage disequilibrium (LD) clumping was then performed using the European population from the 1000 Genomes Project Phase 3 as the reference panel, with parameters set at *r* ^2^ = 0.001 and a window size of 10,000 kb, to minimize LD. Subsequently, exposure and outcome datasets were harmonized by excluding SNPs with allele mismatches or those forming palindromic sequences with intermediate frequencies. To address potential reverse causality, the Steiger test was employed to remove SNPs where the causal direction might be confounded. To reduce bias from weak IVs, we retained only SNPs with an F‐statistic greater than 10. Finally, SNPs associated with confounding variables were excluded from the analysis.

#### 2.2.5 Univariable MR Analysis

UVMR analyses were executed in R software (version 4.2.2) using the Two‐sample MR package (v0.5.6) and MRPRESSO toolbox (v1.0). Causal relationships between genetically predicted exposures and outcomes were primarily assessed through the inverse‐variance weighted (IVW) method (Spiga et al. 2023). Complementary methods, including MR‐Egger regression, weighted median, weighted mode, and simple mode estimator, were applied to ensure robustness and address potential pleiotropy (Bowden et al. 2015; Bowden et al. 2016). Statistical significance was defined as false discovery rate (FDR)‐adjusted *p* values < 0.05, with correction applied to IVW‐derived estimates. Power calculations for binary outcomes utilized an online computation tool. In forward MR analyses, odds ratios (ORs) quantified sleep disorder risk per standard deviation (SD) increased in brain rsfMRI phenotypes, where OR = 1 denotes neutral risk, OR < 1 indicates risk reduction, and OR > 1 signifies elevated risk.

#### 2.2.6 6 Sensitivity Analysis

A series of sensitivity analyses were conducted to assess the robustness of the findings and identify potential pleiotropic effects. Heterogeneity between IVs was tested using Cochran's *Q* statistic, defining a *p* value < 0.05 as statistically significant (Burgess and Thompson 2017). Horizontal pleiotropy was assessed using the MR‐PRESSO global test (global test) and intercept term (Verbanck et al. 2018), while the Steiger test was applied to validate the direction of causality and exclude reverse causality. In addition, funnel plot asymmetry was examined to detect systematic heterogeneity or publication bias by visualizing the distribution of variant‐specific estimates against their precision. Leave‐one‐out analysis determined result stability by sequentially excluding individual SNPs and recomputing effect estimates, identifying disproportionately influential variants.

#### 2.2.7 Multivariable MR Analysis

MVMR, a further extension of UVMR, was applied to examine the direct causality between rsfMRI phenotypes and sleep disorders. MVMR was conducted to identify potential confounders. MVMR incorporates potential confounding factors into the model to eliminate their influence. If the relationship between exposure and outcome remains robust, it can be inferred that the effect is independent of confounding factors. Smoking, alcohol consumption (Liu et al. 2019), tea intake, coffee intake, and cannabis use (Pasman et al. 2019), were selected based on their strong genetic correlations with both brain function and sleep disorders. To infer causality, IVW and MR‐Egger methods based on weighted linear regression were used. The analysis was done in R software using the MR (0.5.6) and MVMR (0.3.0) packages.

## 3 Results

### 3.1 Univariable MR Analysis

#### 3.1.1 Forward MR of Brain Resting‐State Functional Networks on Sleep Disorders

Three amplitude‐based phenotypes demonstrated potential causal relationships with two specific sleep conditions: daytime napping and OSA. These associations reached statistical significance at FDR‐corrected thresholds (q < 0.05).

#### 3.1.2 Effects of Brain Resting‐State Functional Networks on Daytime Napping

**FIGURE 2:**
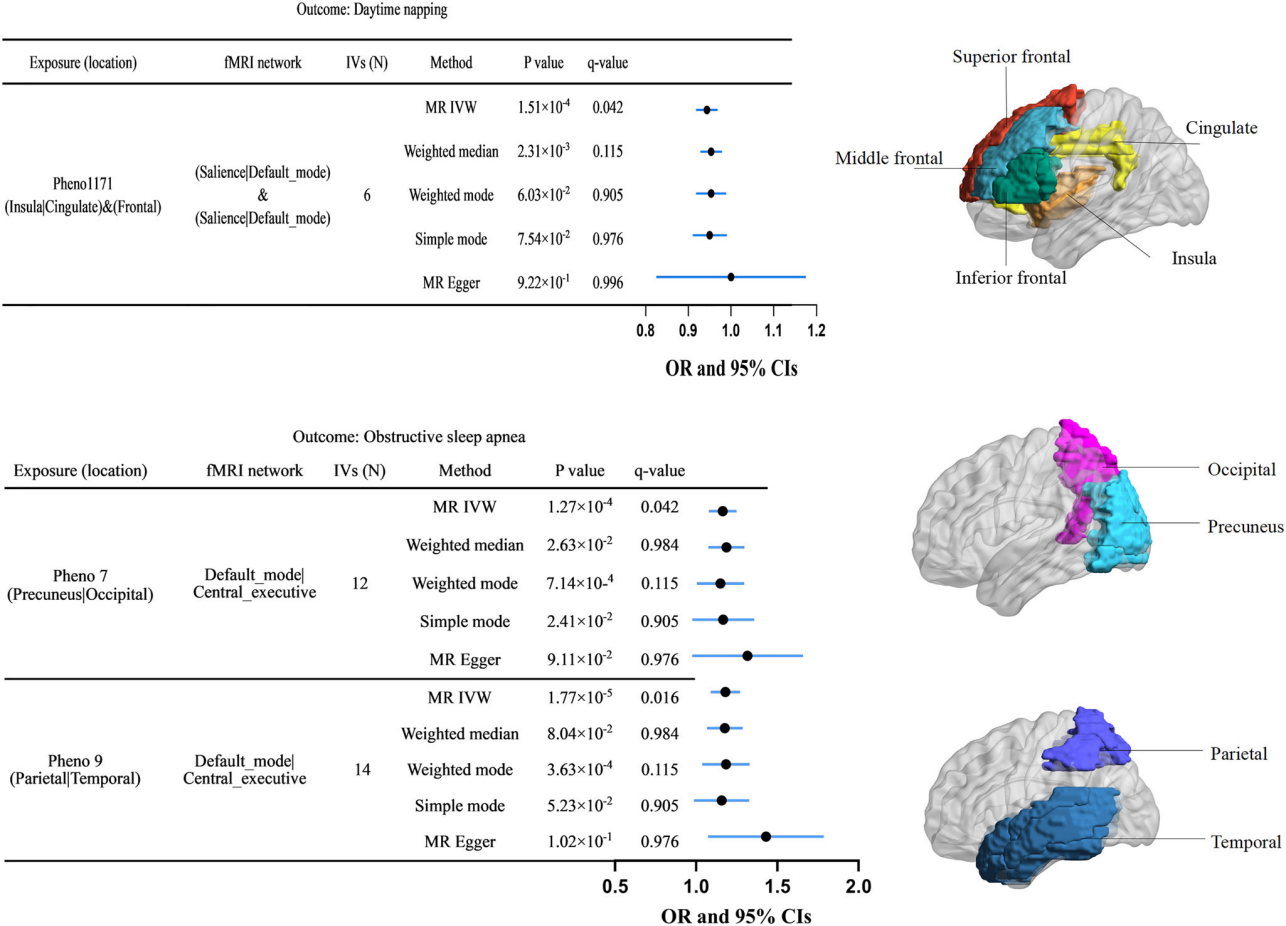
The results of the forward MR analysis. Right: pattern diagram showing the brain anatomical region of the corresponding rsfMRI phenotypes. Left: The forest plot of two‐sample MR analyses for the effect of brain resting‐state functional activities on risk of sleep traits. Each solid circle represents the effect (i.e., OR change) per 1 SD change in the respective brain resting‐state functional activities on sleep traits, and the error bars represent 95% CI. CIs, confidence intervals; fMRI, functional magnetic resonance imaging; IVs, instrumental variables; IVW, inverse variance weighted; MR, Mendelian randomization; OR, odds ratio.

Forward MR analyses indicated that the amplitude of neural activity of the insular or cingulate and frontal regions were negatively associated with daytime napping risk (Figure 2, Table S4). The activity of these brain regions affected the functional connectivity in the DMN or SN, and 1 SD increases in the functional connectivity of these networks was associated with 6% lower risk of daytime napping. IVW OR = 0.94 (95% CI: 0.92–0.97; pFDR = 0.042), MR‐Egger OR = 0.99 (95% CI 0.83–1.18, *p* = 9.92 × 10^−1^), weighted median OR = 0.95 (95% CI 0.93–0.98, *p* = 2.31 × 10^−3^), weighted mode OR = 0.95 (95% CI 0.92–0.99, *p* = 6.03 × 10^−2^), and simple mode OR = 0.95 (95% CI 0.91–0.99, *p* = 7.54 × 10^−2^).

#### 3.1.3 Effects of Brain Resting‐State Functional Networks on Obstructive Sleep Apnea

OSA, characterized by recurrent partial or complete upper airway collapse during sleep leading to fragmented sleep and excessive daytime sleepiness, demonstrated significant associations with two specific rsfMRI phenotypes in this study. These phenotypes were associated with the amplitude of neural activity in the precuneus, occipital, parietal, or temporal regions, which are primarily involved in sensory information processing, cognition, memory, and language functions (Figure 2, Table S4).

Our findings indicated that Pheno7, representing increased amplitude of neural activity in precuneus or occipital regions and interpreted as elevated the DMN or CEN connectivity, was positively associated with OSA. A 1 SD increase in Pheno7 corresponded to 16% higher OSA risk. IVW OR = 1.16 (95% CI: 1.08–1.25; pFDR = 0.042), MR‐Egger OR = 1.40 (95% CI 1.09–1.80, *p* = 2.63 × 10^−2^), weighted median OR = 1.17 (95% CI 1.07–1.29, *p* = 7.14 × 10^−4^), weighted mode OR = 1.18 (95% CI 1.04–1.33, *p* = 2.41 × 10^−2^) and simple mode OR = 1.15 (95% CI 0.99–1.33, *p* = 9.11 × 10^−2^). As shown in Figure 2, within the DMN and CEN, a 1 SD increase in the amplitude trait (node) of the precuneus and occipital regions was associated with a 16% higher OSA risk, as estimated by multiple MR methods. The amplitude trait reflects rsfMRI signal changes, used to assess neural activity intensity in relevant functional areas.

For ​​Pheno9, our MR analyses suggested that the amplitude of neural activity in the parietal or temporal regions at resting state was positively associated with OSA, and 1 SD increase in the functional connectivity of the DMN or CEN increases the risk of OSA by 18%. IVW OR = 1.18 (95% CI 1.09–1.27, *p* = 1.77 × 10^−5^, pFDR = 0.016), MR‐Egger OR = 1.29 (95% CI 0.99–1.67, *p* = 8.04 × 10‐^2^), weighted median OR = 1.18 (95% CI 1.08–1.30, *p* = 3.63 × 10^−4^), weighted mode OR = 1.14 (95% CI 1.01–1.30, *p* = 5.23 × 10^−2^), and simple mode OR = 1.16 (95% CI 0.98–1.36, *p* = 1.02 × 10^−1^) (Figure 2). The effect directions from the four supplementary MR methods aligned with the primary IVW analysis.

#### 3.1.4 Reverse MR of Sleep Disorders on Brain Resting‐State Functional Networks

In the reverse MR analysis, we observed no statistically significant causal effects of sleep disorders on brain rsfMRI after FDR correction. The detailed results of genetic associations are provided in Table S5.

#### 3.1.5 Sensitivity Analysis

The MR‐Egger intercept test indicated no horizontal pleiotropy across associations (*p* > 0.05; Table 2). While heterogeneity was detected for pheno1171 (daytime napping) and pheno9 (OSA) (*p* < 0.05), MR‐PRESSO global testing confirmed that heterogeneity did not bias causal estimates. Steiger directional testing rejected reverse causality (*p* < 0.05). Leave‐one‐out analyses validated result stability (Figure S1), with no single SNP disproportionately influencing outcomes. Forest plots from MR single‐SNP tests (Figure S2) visualized causal effects of individual SNPs, while symmetrical funnel plots (Figure S3) indicated no evidence of publication bias.

**TABLE 2:** The results of sensitivity analysis.

Exposure	Outcome	MR‐PRESSO Global test		IVW		MR Egger		MR‐Egger regression	
		Global.Test	*p*	*Q* test	*p*	*Q* test	*p*	intercept	*p*
Pheno1171	Daytime napping	17.619	0.102	11.091	0.050	10.293	0.036	−0.003	0.607
Pheno7	OSA	16.539	0.280	13.894	0.239	11.329	0.332	−0.013	0.163
Pheno9	OSA	23.177	0.111	19.937	0.097	19.173	0.084	−0.006	0.502

Abbreviations: IVW, inverse‐variance‐weighted; MR‐PRESSO, Mendelian randomization pleiotropy residual sum and outlier; *p*, *p* value; *Q* test, Cochran's *Q* test.

#### 3.1.6 Multivariable MR Analysis

**FIGURE 3:**
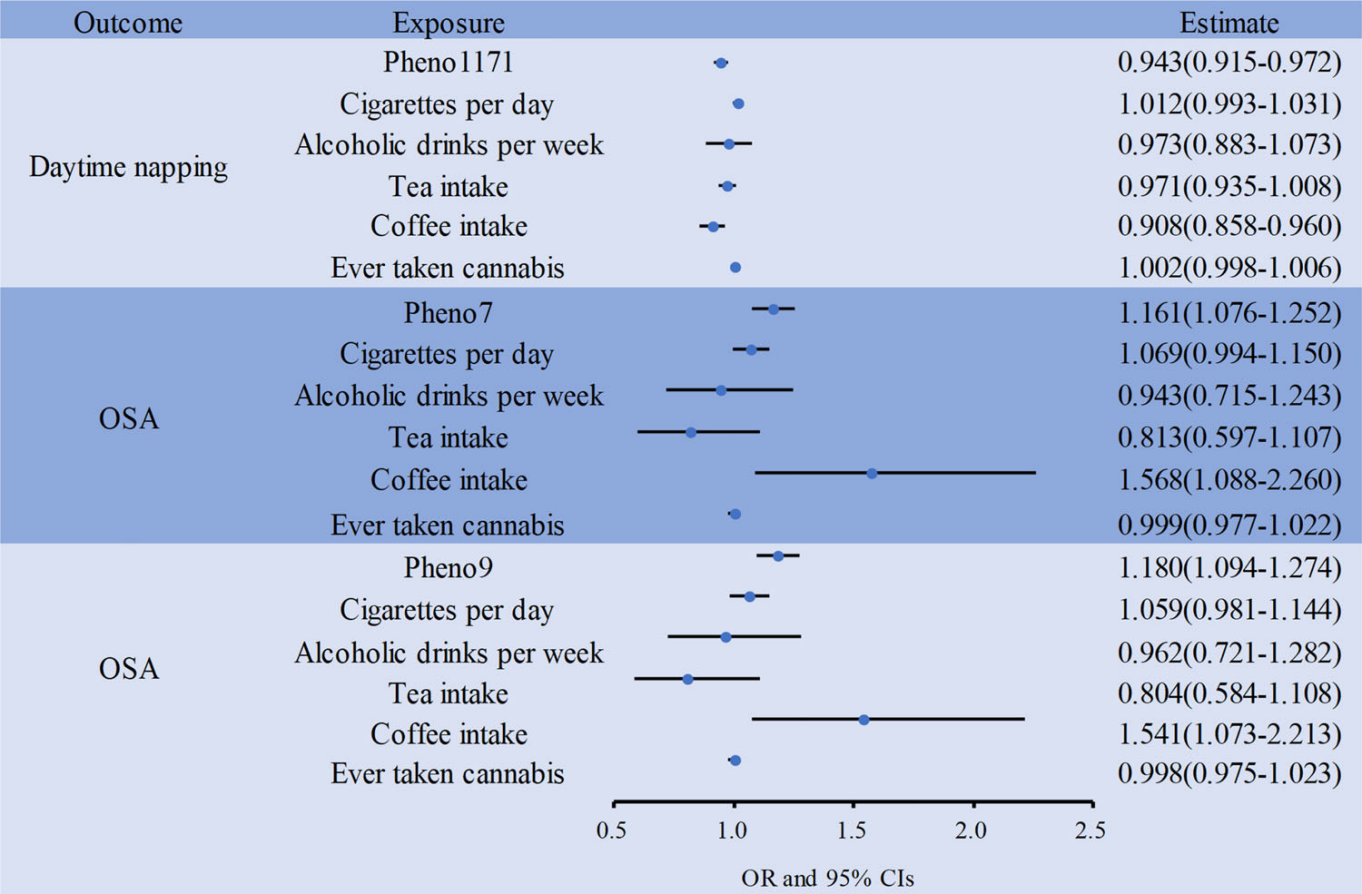
The results of the Multivariable MR analysis. Forest plots of Multivariable Mendelian randomization analyses examined causal associations between rsfMRI phenotypes and sleep traits, adjusted for cigarettes, alcoholic, tea intake, coffee intake, and cannabis. Estimates of causality were expressed as odds ratios (ORs) and 95% confidence intervals (CIs). OSA, obstructive sleep apnea.

MVMR analyses (Figure 3, Table S6) showed that for the association between Pheno1171 and daytime napping, the relationship remained significant only after adjusting for coffee intake (IVW OR = 0.91; 95% CI: 0.86–0.96; *p* = 0.001). In contrast, adjusting individually for smoking, alcohol consumption, tea intake, or cannabis use weakened this association to non‐significance, indicating these four behaviors may act as confounding factors affecting the Pheno1171‐daytime napping link.

For the associations between Pheno7 and OSA, as well as between Pheno9 and OSA, the relationships remained significant after adjusting for coffee intake (Pheno7: IVW OR = 1.57, 95% CI: 1.09–2.26, *p* = 0.016; Pheno9: IVW OR = 1.54, 95% CI: 1.07–2.21, *p* = 0.019). This suggests coffee intake does not substantially confound these rsfMRI–OSA links. Adjusting for other addictive behaviors individually or collectively showed varying degrees of influence on the association strength, but the core significance persisted when accounting for coffee. Multivariable Egger intercept tests showed no horizontal pleiotropy (all *p* > 0.05).

## 4 Discussion

Employing two‐sample MR design, this study systematically evaluated causal relationships across 191 rsfMRI phenotypes and sleep disorders. Key findings identified three specific brain amplitude‐based traits modulating daytime napping and OSA risk, with an observed absence of reverse causality. These mechanistic insights advance our pathophysiological understanding of sleep dysregulation and highlight potential targets for non‐invasive neuromodulation interventions (e.g., transcranial magnetic stimulation) to enhance sleep health.

In our forward MR analysis, we identified Pheno1171, which includes key regions such as the insula, cingulate cortex, and frontal cortex, and is anatomically and functionally associated with the SN and DMN (Menon 2011; Raichle 2015) may act as a protective factor against daytime napping. These networks, particularly the SN, which detects behaviorally relevant stimuli and modulates attentional shifts between sleep and wakefulness, play a crucial role in maintaining alertness throughout the day (Fang et al. 2015). Studies have shown that the enhancement of internal connectivity between SN and DMN is associated with better attention control and a reduction in daytime napping (Fang et al. 2021; Zhou et al. 2023). Similarly, the DMN‐which is active in the resting state and is associated with self‐referential thinking‐helps to maintain arousal and alertness (Corbetta and Shulman 2002). Thus, increased connectivity between these brain regions may reduce the likelihood of daytime napping by improving attentional control and cognitive flexibility (Hickey et al. 2020). This finding supports the hypothesis that efficient information processing within these neural networks functions as a neuroprotective mechanism, thereby reducing the need for excessive daytime napping.

In contrast, amplitude‐based Phenotypes 7 and 9, reflecting heightened neural activity in key regions of the DMN and CEN, are associated with an increased risk of OSA. Specifically, Pheno7 captures increased activity in precuneus or occipital regions, while Pheno9 reflects elevated activity in parietal or temporal regions. These regions are critical for visual processing, spatial perception, and sensory integration. Dysregulation of neural activity in these hubs may impair the brain's ability to regulate breathing during sleep, a key factor in the pathogenesis of OSA (Xie et al. 2022). CENs involved in higher‐order cognitive functions may contribute to the executive dysfunction at the core of OSA pathogenesis (Huang et al. 2023; Zhang et al. 2013) demonstrated that dysfunctional connectivity in regions such as the prefrontal cortex, which is a core component of the CEN, is associated with impaired respiratory muscle control during sleep. This suggests that altered neural network connectivity, particularly in brain regions responsible for sensory processing and cognitive control, may play a key role in the onset and progression of OSA.

No significant reverse causality was found after FDR correction, suggesting that the influence of functional brain networks on sleep disorders may far outweigh the influence of the latter on the former. Although sleep characteristics may also influence brain network connectivity, our results suggest that brain functional connectivity plays an important role in shaping sleep behavior. This is consistent with findings from longitudinal studies that suggest that alterations in functional connectivity of the DMN precede the onset of sleep disorders (Fasiello et al. 2022; Y. Wang et al. 2023).

Furthermore, our MVMR analysis revealed that factors such as cigarette smoking, alcohol consumption, tea, and cannabis use ​​act as significant confounders​​ in the relationship between brain networks and sleep disorders. Addictive behaviors are known to alter brain connectivity in ways that could exacerbate sleep disorders. For example, nicotine and alcohol disrupt connectivity within the SN, impairing attentional shifts (K. S. Wang et al. 2021). After adjusting for these ​​potential confounders​​, we observed significant alterations in the association between certain rsfMRI phenotypes and sleep disorders, suggesting that addictive behaviors ​​contribute to spurious associations​​ between neural phenotypes and sleep outcomes (Galandra et al. 2018). These findings highlight the importance of considering addictive behaviors as ​​key confounding variables​​ in the relationship between the brain function and sleep health. Future research should explore the mechanisms through which addictive behaviors ​​operate as confounders​​ and investigate potential interventions that target both brain function and addictive behaviors to improve sleep outcomes.

This study has several limitations. First, the GWAS datasets utilized were predominantly obtained from European populations, requiring future validation in other ethnic groups. Second, almost all existing MR methods need to meet several basic assumptions. For the gene–environment equivalence assumption (Sanderson et al. 2022), despite the effects of genetic variation on exposure being usually similar to those of the environment, genetic variation is unlikely to fully mimic environmental changes, which may also lead to some bias in our interpretation of the results. Finally, although we identified putative causal relationships between dysregulated network connectivity and sleep disorders, these predominantly statistically derived associations necessitate confirmation through longitudinal clinical studies.

## 5 Conclusion

Overall, the research suggests that specific brain rsfMRI phenotypes are causally linked to sleep disorders such as daytime napping and OSA. The findings indicate that the intensity of neural activity in certain brain regions, including the insula, cingulate, frontal cortex, and others, as well as the functional connectivity between the SN, the DMN, and the CEN, play significant roles in modulating the risk of these sleep disorders. In addition, the study highlights that smoking, alcohol consumption, tea intake, and cannabis use may act as confounding factors in the relationship between rsfMRI phenotypes and sleep disorders. These results may help identify new therapeutic targets and intervention strategies to improve sleep health and address sleep disorders.

## Author Contributions

**Wenting Lin**: conceptualization, data curation, investigation, writing – original draft, writing – review and editing, formal analysis, methodology. **Xiaoqing Chen**: project administration, writing – review and editing, validation. **Yuxing Wei**: methodology, visualization. **Lu Yang**: software, data curation. **Hui Li**: supervision, formal analysis, project administration. **Tianmin Zhu**: supervision, formal analysis, resources, funding acquisition.

## Conflicts of Interest

The authors declare no conflicts of interest.

## Peer Review

The peer review history for this article is available at https://publons.com/publon/10.1002/brb3.70870.

## Supporting information




**Supplemental Figure. 1. Leave‐one‐out analysis diagram of the causal relationship between** rsfMRI phenotypes **and sleep traits in MR analysis**. The X‐axis corresponds to the log (ratio) effect of exposure on outcome. A, phenotype 1171 on the risk of daytime napping; B, phenotype 7 on the risk of OSA. C, phenotype 9 on the risk of OSA. OSA, obstructive sleep apnea.

**Supplemental Figure. 2. Forest plots of** rsfMRI phenotypes**‐associated SNPs on sleep traits risk in the Single SNP test**. A, phenotype 1171 on the risk of daytime napping; B, phenotype 7 on the risk of OSA. C, phenotype 9 on the risk of OSA. OSA, obstructive sleep apnea.

**Supplemental Figure. 3. Funnel plots for the causal estimates of** rsfMRI phenotypes **on sleep traits. Each dot represents an SNP as a genetic tool**. A, phenotype 1171 on the risk of daytime napping; B, phenotype 7 on the risk of OSA. C, phenotype 9 on the risk of OSA. OSA, obstructive sleep apnea.

**Supplementary Tables**: brb370870‐sup‐0004‐TableS1‐S6.xlsx.

## Data Availability

GWAS summary statistics for 191 brain resting‐state fMRI phenotypes are accessible via Zenodo (https://zenodo.org/record/5775047). Sleep disorder and addictive behavior datasets originate from UK Biobank (https://www.ukbiobank.ac.uk), while obstructive sleep apnea data derive from FinnGen (https://www.finngen.fi/en). Metrics for cigarettes per day and alcoholic drinks per week were obtained from GSCAN (https://genome.psych.umn.edu/index.php/GSCAN).
