# Supplementary figures and images for: Causal Relationship Between Brain Functional Networks and Sleep Disorders: A Mendelian Randomization Study
Source: Brain Behav. 2025 Oct 7;15(10):e70870. doi: 10.1002/brb3.70870 (PMC12504808; doi:10.1002/brb3.70870)

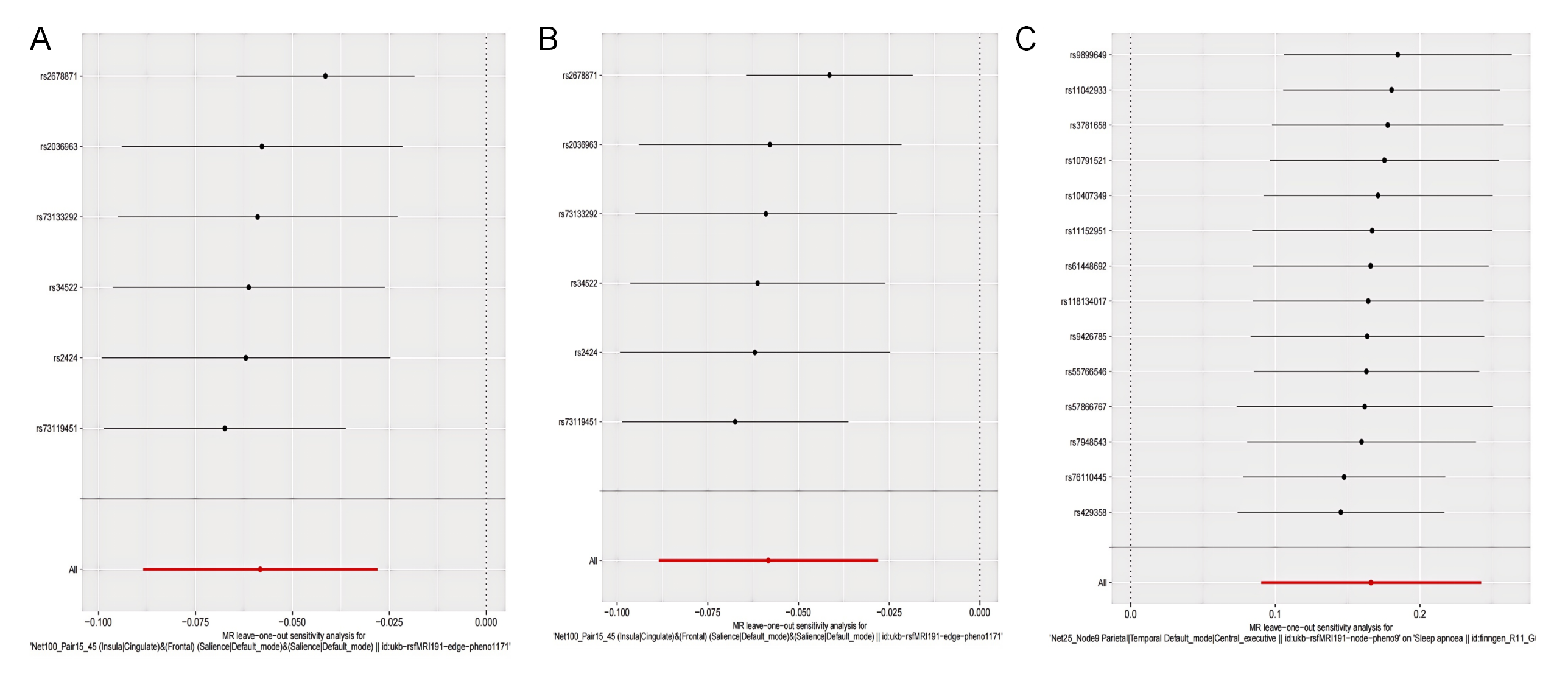

Supplement: Supplementary file 1 — Supplemental Figure. 1. Leave‐one‐out analysis diagram of the causal relationship between rsfMRI phenotypes and sleep traits in MR analysis. The X‐axis corresponds to the log (ratio) effect of exposure on outcome. A, phenotype 1171 on the risk of daytime napping; B, phenotype 7 on the risk of OSA. C, phenotype 9 on the risk of OSA. OSA, obstructive sleep apnea. [file BRB3-15-e70870-s003.tif]

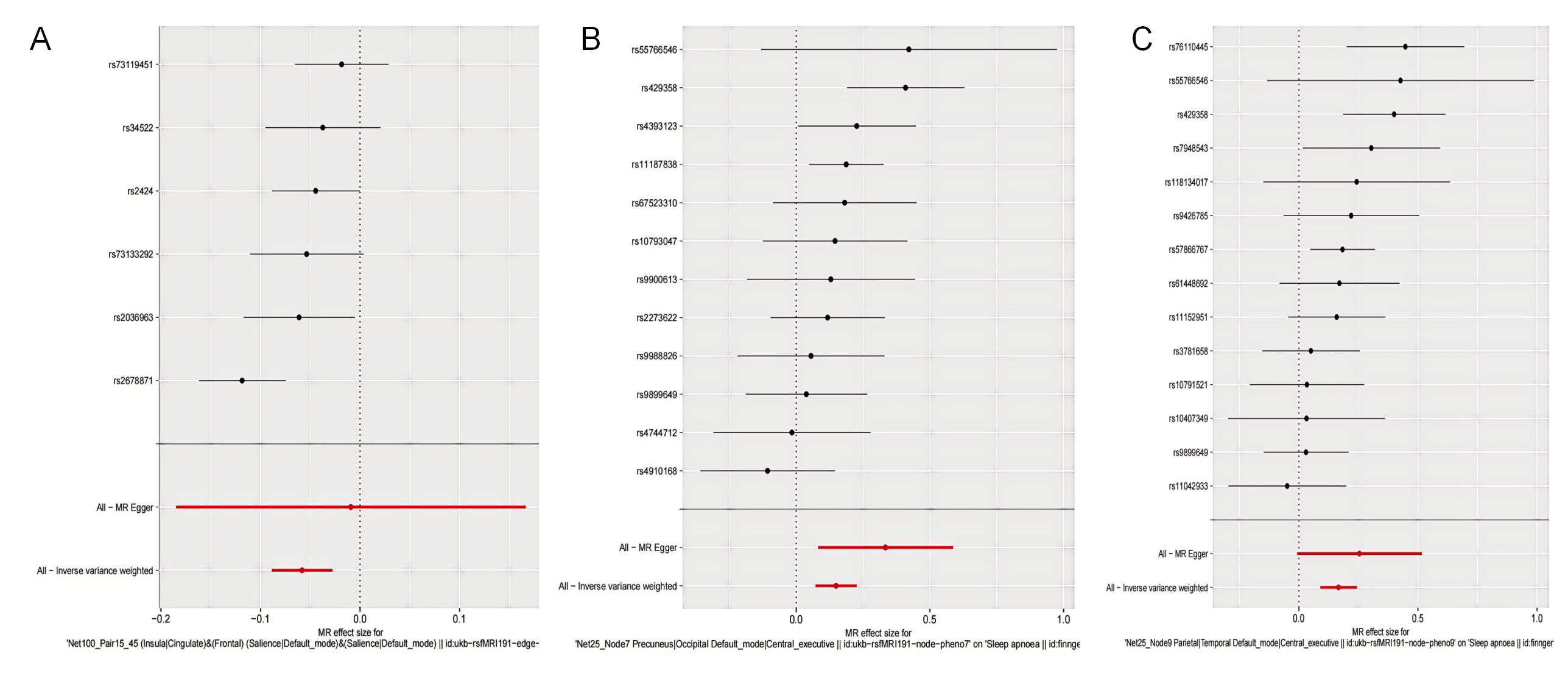

Supplement: Supplementary file 2 — Supplemental Figure. 2. Forest plots of rsfMRI phenotypes‐associated SNPs on sleep traits risk in the Single SNP test. A, phenotype 1171 on the risk of daytime napping; B, phenotype 7 on the risk of OSA. C, phenotype 9 on the risk of OSA. OSA, obstructive sleep apnea. [file BRB3-15-e70870-s004.tif]

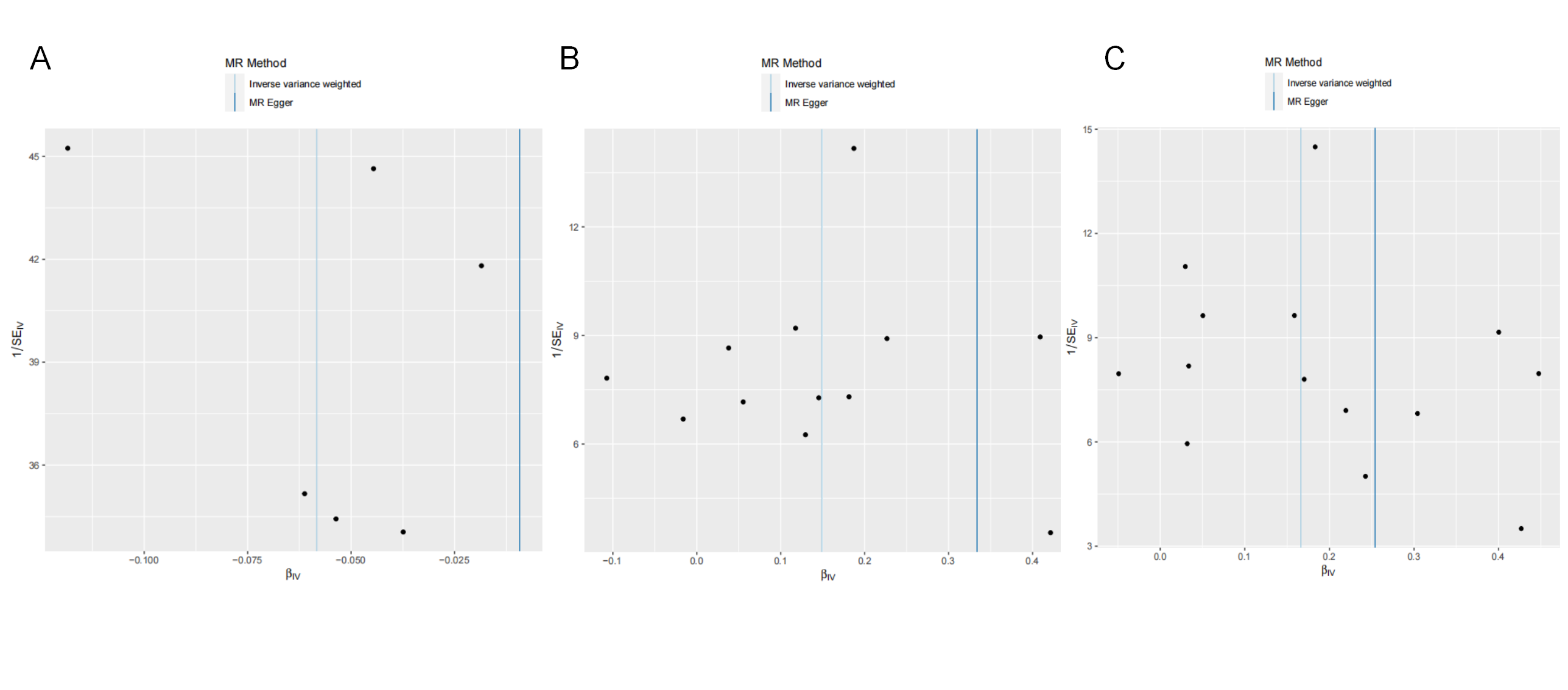

Supplement: Supplementary file 3 — Supplemental Figure. 3. Funnel plots for the causal estimates of rsfMRI phenotypes on sleep traits. Each dot represents an SNP as a genetic tool. A, phenotype 1171 on the risk of daytime napping; B, phenotype 7 on the risk of OSA. C, phenotype 9 on the risk of OSA. OSA, obstructive sleep apnea. [file BRB3-15-e70870-s001.tif]
